# 46920 Engagement and co-creation in a DNA integrity cohort study

**DOI:** 10.1017/cts.2021.597

**Published:** 2021-03-30

**Authors:** Martha I. Arrieta, L. Lynette Parker, Chantel M. Bonner, Robert W. Sobol

**Affiliations:** 1 University of South Alabama Center for Healthy Communities; 2 USA Health Mitchell Cancer Institute & Department of Pharmacology, University of South Alabama

## Abstract

ABSTRACT IMPACT: Our work is demonstrative of the value embedded in community engagement as a vehicle to facilitate and expand the focus of translational research. OBJECTIVES/GOALS: To develop a community-informed recruitment process for a population-based DNA integrity longitudinal study aiming to document the average amount of DNA damage as well as DNA repair capacity in a cohort of community-dwelling individuals. METHODS/STUDY POPULATION: The three-member Community Engagement team (CE Team) partnered with a ten-person Community Advisory Board (CAB) to develop recruitment procedures and materials. Through an iterative process taking place over 13 meetings, CAB members answered questions about community context, appropriate recruitment approaches, and tone of communication with potential study participants. They also collaborated in the creation of outreach materials, informational booklets, and the informed consent document. The CAB’s input was recorded in meeting notes that informed successive versions of the materials. The CE Team held post-meeting debriefs to develop consensus on lessons learned and next steps. RESULTS/ANTICIPATED RESULTS: CAB input generated a five-step recruitment process. It informed approaches to communications with potential participants and resulted in a set of printed recruitment materials. Furthermore, the CAB pushed the CE Team and laboratory scientists to think beyond study participation to a comprehensive view of respectful engagement including notification of elected officials and other community institutions. By sharing personal anecdotes and asking how this study would reflect their lived experiences and/or contribute to their communities, CAB members inspired the university team to recognize the environmental context that may underlie DNA damage in residents of an underserved community. DISCUSSION/SIGNIFICANCE OF FINDINGS: The CAB was very effective in generating tools for recruitment. Moreover, CAB members provided insights beyond those originally sought by the CE Team, regarding broader engagement and a focus of future research relevant to the needs of both the community and the university researchers.

